# Women’s decision process when actively choosing to ‘go flat’ after breast cancer: a constructivist grounded theory study

**DOI:** 10.1186/s12905-024-03015-0

**Published:** 2024-03-15

**Authors:** Anna Paganini, Linda Myrin Westesson, Emma Hansson, Susanne Ahlstedt Karlsson

**Affiliations:** 1 Region Västra Götaland, Department of Plastic and Reconstructive Surgery, Sahlgrenska University hospital, Gothenburg, Sweden; 2 Sahlgrenska Academy, Institution for Health and Care Sciences at Gothenburg University, Gothenburg, Sweden; 3 Sahlgrenska Academy, Institution for Clinical Sciences at Gothenburg University, Gothenburg, Sweden; 4 Region Västra Götaland, Department of Medicine, Sahlgrenska University Hospital, Gothenburg, Sweden; 5 Region Västra Götaland, Department of Surgery, Sahlgrenska University Hospital, Gothenburg, Sweden

**Keywords:** Breast cancer, Breast reconstruction, Flat closure, Mastectomy, Decision making, Patient experience, Grounded theory

## Abstract

**Objective:**

This study aims to describe a conceptual model that could illuminate the decision process women go through when choosing to go flat on one or both sides due to breast cancer.

**Methods:**

A qualitative design, with constructivist grounded theory was used. Eighteen women were individually interviewed, digitally or by telephone, until saturation was reached. Data were analysed using a constant comparative iterative method in accordance with grounded theory. By examining the text data to identify the decision process for going flat and rejecting reconstructive surgery open coding was obtained. As the study proceeded patterns were explored and categories developed into a core category.

**Results:**

The overall decision process for women choosing to go flat on one or both sides emerged in three phases: Phase 1, where the women are forced to “Face the cancer”, Phase 2 comprising “Reflections on health and motivation” and Phase 3, described as “Hobson’s choice”. The fundament of the decision process was found in the core category “Establishing and safeguarding the chosen self”.

**Conclusions:**

The decision process involved in actively going flat and rejecting reconstructive surgery is founded in the individual woman’s motivations, such as view of femininity and apprehensions about the offered reconstructive surgery.

## Introduction

Breast cancer is a prevalent and life-altering disease affecting millions of women worldwide and surgery is an important part of the treatment [1]. In cases where a mastectomy has been performed, women have traditionally been offered an external prosthesis or a breast reconstruction to achieve symmetry in a bra [2]. However, a growing number of breast cancer survivors are choosing a different path and opting for a flat chest on one or both sides [3–6]. Nevertheless, research concerning experiences of flat closure is limited. Women opting for a flat closure are often grouped together with all women who have not had a breast reconstruction, including women on the waiting list for a breast reconstruction and women who want a breast reconstruction but are not candidates for reconstructive surgery [7]. This mix of women makes the evidence base scarce and the patient-care giver dialogue complicated [7, 8]. Some studies with a mixed population [8], have indicated that a bilateral mastectomy leads to an increase in post-operative complications [9] and a decrease in quality of life, as well as body-image [10]. However, the latter can be considered an expected outcome in women who want a breast reconstruction but cannot have it.

In addition, the number of contralateral risk-reducing mastectomies without medical justification has surged, without any medical benefit for the patient [10–12]. Social media and web questionnaires have revealed that major motivations for a flat closure do not include a fear of cancer recurrence [12], but rather the drive towards a healthier body by avoiding implantation of foreign materials, i.e. breast implants, and decreasing the risk of health impairment and surgical complications, thus resulting in a shorter recovery time. This is further combined with the belief that the breasts are unimportant. A previous study has reported that 74% of all women opting for flat closure are satisfied with their decision [13].

The two parallelly developed and distinct phenomena of women who opt to go flat have created a demanding situation for both patients and caregivers. There is a concern that when patients are first diagnosed with cancer and in the initial shock phase, they might make hasty, unfounded decisions based on fear that they will later regret and will result in extensive surgery and morbidity without any medical benefit. This has made some surgeons reluctant to routinely offer a bilateral flat closure [2]. Indeed, women who have opted for a flat closure describe having to battle with their surgeon in order to go flat [6]. In summary, there is a knowledge gap regarding how to distinguish between the two groups in order to better help the women make the right choices regarding treatment and surgery.

Knowledge regarding why women actively choose a flat closure is scarce. Thus, more insight into their decision-making process and the resulting consequences is needed to ensure flat closure can be offered in the correct situations and for the right reasons [7], enabling it to become a fully-fledged alternative in the breast cancer treatment armamentarium. A fundamental prerequisite for shared decision making (SDM) is that patients have self-efficacy, meaning they believe in their own capacity [14]. Thieme et al. (2017) have described that high self-efficacy levels increase cancer patients’ ability to adapt and manage their situation [15]. Self-efficacy is closely related to empowerment, which is a process of gaining strength, confidence and understanding in order to find solutions to a problem [16]. Empowerment can therefore be used to increase patients’ autonomy and participation in their care [17], enabling them to identify which strategies result in feeling empowered and in control [18, 19]. Peer support groups are found to be an important feature in the empowerment process among breast cancer patients [20].

## Objective

In light of the aforementioned perceived need for flat closure experienced by some women with breast cancer and the knowledge gap regarding this choice and its consequences, a conceptual model would be helpful in illuminating the decision process and enabling SDM when women choose to go flat on one side or both due to breast cancer. For the purpose of this study, we have defined ‘go flat’ as the option to have a mastectomy on one or two sides instead of a mastectomy and reconstructive surgery.

## Method

### Qualitative approach, research paradigm and study design

An inductive qualitative design with constructivist grounded theory (CGT) was used [21]. This method underlines the relationship between the participants and the researcher and their interaction throughout data collection, analysis and theory construction [21]. In line with classic GT method, CGT is not a linear process and simultaneous data collection and data analysis was undertaken to establish a theoretical model [21, 22]. The recommendations for qualitative research according to the COREQ criteria were followed throughout the research process [23].

### Ethics approval and consent to participate

The study was approved by the Swedish Ethical Review Authority (2022-02797-01).

All data was treated in accordance with the General Data Protection Regulation [24]. The Helsinki Declaration and Good Clinical Practice guidelines were followed [25]. All participants gave their oral and written informed consent to participation and publication of the results.

### Researcher characteristics and reflexivity

All researchers have a PhD and are clinically active as nurses, or surgeon. The researchers all had prior experience of qualitative research, as well as GT (L.MW). The only researcher who might have had a doctor/nurse-patient relationship with any of the participants (EH) did not participate in data collection and was blinded regarding the identity of participants. Reflexivity was maintained as the interviews were evaluated by three of the researchers (A.P; L.MW; SAK) to recognize potential bias, and any preconceptions were stated before the study. Moreover, looking at the data for competing conclusions further strengthens the results. Reliability and consistency of the analytic procedure was ensured with a transparent research process [26]. To avoid bias, an inductive analysis method was used, and the transcribed interviews and memos were read by several researchers (A.P; L MW; SAK). The codes, subcategories and categories were discussed iteratively until consensus was reached during the research process [27]. The participants were given opportunity to give feedback on the theoretical model.

### Context, participants, and sampling strategy

The study was conducted in Sweden, a country with a publicly financed welfare health care system. The current national guidelines [2] state that all women having a mastectomy have the right to information on both immediate and delayed breast reconstructive surgery. Risk-reducing contralateral mastectomies are only performed in mutation carriers with an increased calculated risk of breast cancer. Regarding contralateral mastectomy for symmetrizing reasons, the guidelines state that it should be performed only after carefully considering the risk of complications and the risk of delaying adjuvant cancer treatment [2].

Participants were recruited through the Swedish patient organisation “Plattnormen” and through the Swedish Breast Cancer Association “Johanna”. The patient organisations reached out to their members and sympathisers and brokered information through social media. Women who wanted to be included contacted the researchers themselves. Participants were eligible if they were above 18 years of age, had been diagnosed with breast cancer and had undergone mastectomy on one or both sides. The women were strategically selected in line with the methodology and following the principles of purposeful sampling [21].

### Data collection

A total of 58 women contacted the research team and expressed a wish to participate in the study. Out of the 58 women, 18 were enrolled before saturation was reached (Table 1). The data were collected between November 2022 and March 2023 from a total of 18 interviews with a mean duration of 48 min (Table 1). The women could choose to be interviewed by telephone (*n* = 3) or the teleconferencing system Zoom© or Teams© (*n* = 15) [28]. All interviews were conducted by AP and SAK, audio recorded and transcribed verbatim. The women were interviewed in their homes or place of choice. The interviews began with an open-ended question: “Tell me about your decision to go flat.” Further questions were developed from earlier interviews to deepen the previously initiated analysis and to ensure focus on the aim of the study in line with CGT [21]. There was a continuous process of analysis and collection of data until saturation was reached and no new insights on the decision process were identified.

**Table 1:** Characteristics of participants

Characteristics of participants (*n* = 18)	Range (median)
Age in years	34–70 (46)
Year since cancer surgery	1–12 (4)
	**n**
Place of care	
County hospital	10
University hospital	8
Type of surgery	
Unilateral mastectomy	5
Bilateral mastectomy	13
Family situation	
Married/Cohabiting	13
Single	4
Missing	1
Education	
Elementary school	0
Upper secondary school	4
College/University	14
SEI ^†^	
Upper SEI	2
Middle SEI	12
Lower SEI	4
Living in	
Rural area	6
Urban area	6
Large city	6
Born outside Sweden	1

†Swedish Socio-economic Classification [XXX] upper SEI: entrepreneurs, lawyers and physicians; middle SEI: civil servants and teachers, and lower; SEI: unskilled and skilled workers; Ref: Swedish Socioeconomic Classification. Stockholm: Statistics Sweden, 1995

### Data analysis

Data were analysed using the constant comparative method, which is a process whereby data collection and analysis are carried out simultaneously and iteratively [22]. Throughout the process, memos were written directly after every interview. As categories emerged, further memos were written to enable detailed comparisons [21]. Investigator triangulation was achieved, as all the researchers were active in the analysis process [29, 30]. Open coding was reached by examining the text data to identify the decision process involved in going flat and rejecting reconstructive surgery. As the study progressed, patterns were explored and categories developed into a core category and a theoretical model.

## Results

**Fig. 1:**
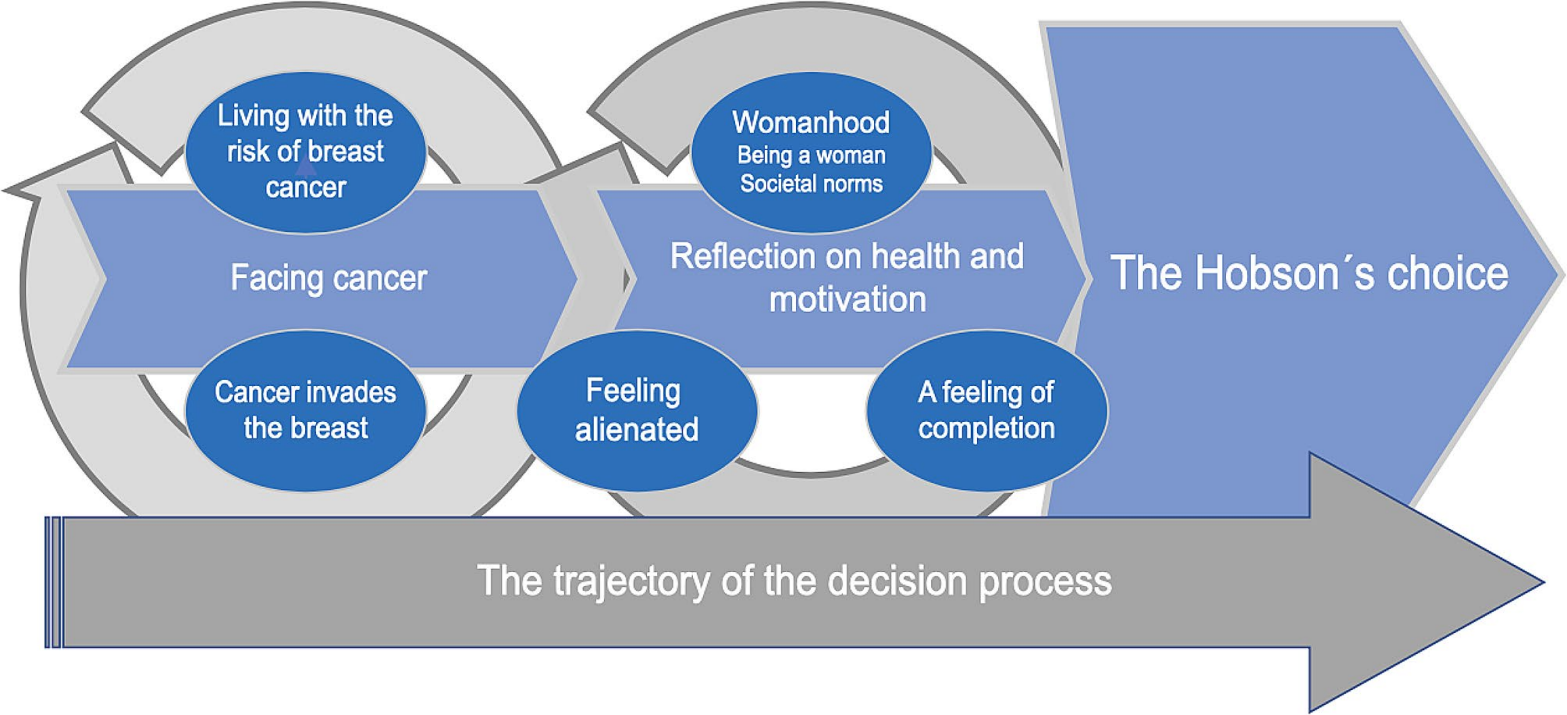
Core category: establishing and safeguarding the chosen self

The overall process for women choosing to go flat on one or both sides is reflected in the core category: “*Establishing and safeguarding the chosen self”*. A linear process with three phases was identified. Identifying these three phases was necessary, as it created an opportunity for the women’s self-reflection on body and health. All the participants were found to have experienced all three phases, although individuals differed in terms of how long they spent in each phase. The first phase included being forced into *“Facing cancer”* when diagnosed with breast cancer or with a gene mutation causing increased risk of developing a breast cancer. The second phase comprised *“Reflections on health and motivation”*, where the women processed both the diagnosis of breast cancer and the offer of reconstructive surgery. Both are acknowledged and described with feelings of alienation. Moreover, as the participants did not feel their womanhood was affected due to the lack of one or two breasts, a third phase emerged: *“Hobson’s choice”*, or a non-choice. Through inductive, iterative analysis the core category emerged and was described as the women seeking completion as an endeavour to obtain health and wellbeing (Fig. 1). A description of the three phases underpinning the theoretical model is illustrated with quotes from women who actively chose to go flat on one or on both sides.

### Phase 1 – Facing cancer

The decision process starts with facing cancer and being forced to take action, due to, for example, being diagnosed with breast cancer or with a genetic mutation. The process might also start if a family member, such as a mother, grandmother or sister, has been diagnosed and subsequently treated for breast cancer. In this first phase two subthemes are identified and described:

“*Cancer invades the breast*” and * “Living with the risk of breast cancer”*

#### Cancer invades the breast

This subcategory emerged when a breast cancer was diagnosed. The breast cancer itself was fear inducing and acknowledged as something foreign and frightening to the body and to the women’s very being. This caused feelings of estrangement to the breast, and a wish to remove it in order to feel safe again, regardless of the tumour size.

And I know that I had kind of, my first thought was like, I just want to remove this…(P11, 37 years)

Once diagnosed, feeling invaded by a breast cancer forced the women to take action, i.e. surgery, in order to survive. During this first phase of the decision process the women felt that action had to be taken quickly, without time to reflect. When looking back on this time period, many women described it as traumatizing. The need to make a critical decision without opportunity for reflection created a sense of chaos.

…but everything also went so fast that you kind of didn’t have time, you were just thrown around like in some tombola machine. (P15, 45 years)

#### Living with the risk of breast cancer

Many women described living with the risk of breast cancer for many years, long before their diagnosis. In many cases they had encountered cases of cancer in their family and lived in fear of having the same experiences. This fear also included relapse or surviving the disease but living with the consequences afterwards.

And yes, since she [mother] got her breast cancer, I’ve basically been prepared since the age of 25 and seen myself as if I’m going to get breast cancer (P 18, 62 years)

Some women had a diagnosis of a gene mutation with increased risk of breast cancer, which included them in a program with extra check-ups at regular intervals, leading to additional stress. The gene mutation was perceived as a threat to their health and future and was fear inducing.

But then I found out that I had the gene [BRCA-gene mutation] and then they also started with MRI [Magnetic Resonance Imaging] so I had time to go for it twice … and because these MRI checks caused a lot of concern and it took a lot of time, I thought, unnecessary worry … (P 14, 34 years)

Moreover, following diagnosis and breast cancer surgery, fear of a cancer relapse influenced the wish for further surgery to decrease the risk with a contralateral mastectomy.

### Phase 2 – Reflections on health and motivation

The second phase, reflection on one’s health situation and personal motivations, revealed how the women reflect on and create the basis for the final decision to reject reconstructive surgery. This phase involved feelings of estrangement caused by breast cancer and formulated the goal of being healthy by having a body free from foreign materials and not being subjected to further large surgeries. The women also described how they reflected on societal norms and on being a woman in relation to having breasts. This phase included three categories, “*Feeling alienated”*, “*A feeling of completion”* and “*Womanhood”*, and was described as lasting for weeks or even years, as the women needed time to reflect on their motivations.

#### Feeling alienated

The women described feelings of estrangement, as the reconstructed breast offered was considered to be non-authentic and therefore a barrier to feeling healthy. Many women had insecurities regarding the prothesis itself, as they were afraid the material would be carcinogenic. They also expressed a fear that a prosthesis would hide a relapse. Another major concern was the potential loss of sensation in the reconstructed breast, the permanent prothesis feeling non-authentic and lacking the features that define a natural breast. This caused the women to feel they were losing a bit of their relationship with the breast, as the prosthesis was considered foreign and alien to the body.

because I got these implants first, and it’s not nice, I promise you. Nothing feels like your own. It doesn’t feel like it’s your own boob, it, like… you don’t feel it… and it feels… it felt very foreign in my body. (P 8, 42 years)

#### A feeling of completion

During this middle phase of the decision process the women said they finally wanted to feel healthy again and were consequently not motivated to undergo reconstructive surgery with all the risks, burdens and potential problems that might occur. The women also wanted the cancer process to come to an end in order to move forward in life with a sense of safeguarding themselves.

They told me, since I was going to have radiation afterwards, you would have to wait, then you would have to remove the belly and back and do something like that… DIEP flap or whatever it’s called… and then I felt like this, a lot of operations again … on your already scarred body in some way and then I felt that what I’m getting is not what I had, so … I’m not getting my old breast back, I get another one that doesn’t have any feeling and doesn’t, or like it’s not the same at all. (P 5, 45 years)

#### Womanhood

The third category in the second phase includes the women’s view of a female body and its appearance, but also societal norms and how that affects the decision process. The two sub-categories identified were “*Being a woman”* and “*Societal norms”.*

##### Being a woman

The women in the study described how their femininity was not related to their breasts, despite societal norms saying otherwise. Breasts were often described as unimportant to the individual and of little value, and not the defining attribute of being a woman. The view of the breast in relation to being a woman was not something that occurred at diagnosis, but could instead be something the women had experienced throughout their lifetimes. The breast was not regarded as an important feature of their self-image of being a woman.

I’m a woman, girl, lady, whatever label you want to put on it. Never felt like anything else. So why should, like, two fat lumps decide anything at all, it’s kind of irrelevant to me. (P 16, 44 years)

Breasts were described as being closely connected to a woman’s sexuality and some women argued for saving one breast, as breasts are considered an erogenous zone. However, many women reasoned that sexuality was much more than the breasts, and their absence did not affect their sexuality. Rather, it was the hormonal cancer treatment that changed the women’s libido and wish for intimacy with their partners.

##### Societal norms

The other sub-category in the middle phase of the decision process describes how societal norms and the opinions of others have little effect on the decision to go flat. The women were confronted with the opinions of friends, acquaintances and health care professionals (HCPs). This category emphasizes how norms of femininity for a woman’s body were related to the breast, which creates a hurdle for women who do not want reconstructive surgery. The women acknowledged that societal norms state that a woman needs to have two breasts, and they could therefore be considered to be norm breakers.

On many occasions the women had been confronted with looks and glances from both acquaintances and strangers but their experience did not cause feelings of shame, instead there was a realisation that this came from curiosity and lack of knowledge, not malice. However, when health care professionals or significant others gave the women critical looks or opinions, this created frustration, and sometimes both shame and anger.

she [the surgeon] turned to my husband and asked, will you really still love your wife like that because she will be flat… it feels like there is so much of a norm in how a woman should look and how you should be and then that hasn’t really progressed along with developments in society, but you can be what you want (P 14, 34 years)

### Phase 3 – Hobson’s choice

The third and final phase was Hobson’s choice, something that is experienced as a non-choice. In this last phase the women were prepared to come to a decision regarding going flat on one or both sides or having reconstructive surgery. Going through the decision process had led to this final phase in which each woman came to her own decision regarding her own body. The women emphasised that they carefully weighed up their options and made their personal decision without the influence of others. They felt that the only possible way forward to establish and safeguard the chosen self was to go flat on one or both sides. A reconstructive surgery was not an acceptable or a justifiable path. The women said reconstructive surgery was not needed in order to feel healthy or feminine. They also stated that reconstructive surgery could not replace what was once lost and deemed the risks to be too high, thus making it a non-choice, with no acceptable alternative other than going flat. The basis for Hobson’s choice could be found in the previous phases, growing through the fear of cancer and solidifying when the individual processes their personal motivations.

yes, felt pretty immediately that the mastectomy somehow felt natural, straightforward for me. (P 9, 53 years)

### Core category “Establishing and safeguarding the chosen self”

Throughout the research process the core category was identified as the choice of going flat made by the individual woman depending on her personal motivations. The women were forced to take responsibility for themselves as they considered the risks of further surgery and their view of their body and femininity, and ended up with only one possible choice, that of no further major surgery. Importantly, several factors, as described in Phase two, affected the women’s wish not to reconstruct.

Yes, but it was probably partly because I am becoming a new woman… so this is a direct gain from the disease. I’m walking with my head held high today and feel like I’m more painless and flexible than I ever have been. (P 13, 70 years)

The core category highlighted that the choice was made in order to be healthy but still feel as a whole woman again, while protecting herself from both cancer and further interventions.

## Discussion

The objective of this study was to develop a conceptual theoretical model that can describe the decision process when women choose to go flat on one or both sides due to breast cancer. The knowledge gleaned from this study stems from the women being empowered to demand to be included in their own care. Such knowledge can enable SDM to illuminate the decision process and reveals the motivations that guide women’s preferences. It highlights the need to include the individual women’s preferences in care. The need to discuss risks, benefits, and consequences of different options in the context of the person’s life and values has been reported [31]. However, there are identified barriers for SDM which include low socio-economic status, multiple comorbidities, language barriers and past negative healthcare experiences. Patients who do not experience these barriers are more likely to be active and engaged in their care [32]. The women participating in this study can all be described as empowered and seeking SDM. HCP have an important role in patients’ ability to feel confident [19], as they can provide individually adapted information to facilitate empowerment [33]. It is important for patients to be empowered to enable them to make informed decisions and participate in care [33]. During phase two the women emphasized that their feeling empowered was a crucial resource in reflecting on their personal health and motivations. Peer support, as provided by patient networks, may also be a strengthening factor that helps the individuals in their decision process [20].

The results of the present study describe the decision process related to the identified core category: “*Establishing and safeguarding the chosen self*” which constitute the foundation for the individual woman’s choice. The core category highlights the decision process, which is founded in and affected by the individual woman’s preferences, including her view of femininity and personal motivation for or against further surgery. The results emphasize that time is needed throughout this linear process, but in a clinical setting there is often a lack of time to enable SDM [34]. SDM and partnership with the HCP regarding going flat was both expected and fundamental for the participants in this study, which is confirmed by precious studies [3, 6, 8, 33].

This study highlights that fear of cancer is not the dominating driving force for women opting for flat closure and abstaining from reconstruction. Although fear is present in the process, there are other aspects that are more significant for the individual and this finding is in line with current research [3, 6–8]. Furthermore, being over 70 years at diagnosis, increasing comorbidities, radiotherapy and adjuvant chemotherapy have been found as significant factors associated with higher likelihood of opting flat closure [35]. The present study adds to the small, albeit slowly growing research concerning the “going flat” movement and shows that some women strive towards flat closure.

## Study limitations

The recruitment of participants took place via patient networks, and potential selection bias was managed by careful selection of the respondents to achieve as heterogenous group as possible [36]. Additionally, health, femininity and womanhood may vary in different cultural contexts. Despite efforts with selection of participants, the group was rather homogenous with all but one participant from Sweden. Recall bias was a possibility since the time since surgery range from 1 year to 12 years. This is difficult to completely mitigate due to the methodology used, but the results should be seen in the light of this [37]. The outcomes of the present study should be considered as one possible interpretation that contributes to a more profound understanding of the women’s decision process. Nevertheless, the present study is one of few that have investigated flat closure, and further research is needed.

## Clinical implications

This study highlights that some women actively choose flat closure. In order to identify their individual motivators, SDM is crucial and enables individually adapted information about the possible paths after a breast cancer diagnosis. Going flat is the only viable option for some women, as reconstructive surgery cannot be considered for personal reasons. To ensure SDM, individual counselling and including the women as partners in their care is necessary.

## Conclusions

The decision process to actively go flat and reject reconstructive surgery includes the individual women’s motivations, such as their view of femininity and their apprehensions about the offered reconstructive surgery. Another key to understanding the “flat closure”-movement is to find ways of recognizing and distinguishing between the women’s different motivations for having a mastectomy, since the origins and driving force seems to differ. By recognizing and addressing the underlying reasons, HCP can provide care that supports women’s autonomy, promotes well-being, and enhances their overall quality of life.

## Data Availability

The datasets used and analysed during the current study are available from the corresponding author on reasonable request.
